# The association between the *TP53* Arg72Pro polymorphism and colorectal cancer: An updated meta-analysis based on 32 studies

**DOI:** 10.18632/oncotarget.13589

**Published:** 2016-11-25

**Authors:** Xin Tian, Shundong Dai, Jing Sun, Shenyi Jiang, Youhong Jiang

**Affiliations:** ^1^ Molecular Oncology Laboratory of Cancer Research Institute, The First Affiliated Hospital of China Medical University, Shenyang, 110001, PR China; ^2^ Department of Pathology, The First Affiliated Hospital and College of Basic Medical Sciences of China Medical University, Shenyang, 110001, PR China; ^3^ Institute of Pathology and Pathophysiology, Shenyang, 110001, PR China; ^4^ Department of Immunology and Biotherapy, Liaoning Cancer Hospital and Institute, Shenyang, 110042, PR China; ^5^ Department of Rheumatology, The First Affiliated Hospital of China Medical University, Shenyang, 110001, PR China

**Keywords:** TP53, colorectal cancer, polymorphism, meta-analysis

## Abstract

Several previous studies evaluated the association between the Arg72Pro (rs1042522) polymorphism in the *TP53* tumor suppressor gene and colorectal cancer (CRC). However, the results are conflicting. This meta-analysis aimed to shed new light on the precise association between *TP53* variants and CRC. We analyzed 32 published case-control studies involving 8,586 cases and 10,275 controls using crude odd ratios (ORs) with 95% confidence intervals (CIs). The meta-analysis was performed using a fixed-effect or random-effects model, as appropriate. We found that the *TP53* Arg72Pro polymorphism was not significantly associated with CRC risk in the overall population. However, subgroup analysis based on ethnicity revealed an increased risk of CRC among Asians (CC vs. GC+GG: OR=1.22, 95% CI: 1.02-1.45), and similar results were found for rectal cancer (CC vs. GC+GG: OR=1.34, 95% CI: 1.120-1.62). These results suggest that the *TP53* Arg72Pro polymorphism CC genotype may contribute to an increased risk of CRC, especially for rectal cancer and among Asians.

## INTRODUCTION

Colorectal cancer (CRC) is the third most commonly diagnosed cancer in males and the second most commonly diagnosed cancer in females. CRC is also the leading cause of cancer-related death in the Western world, and has exhibited a striking rise in incidence in Asian countries [1–3]. The etiology of CRC is multifactorial, though it is widely accepted that CRC can be caused by an accumulation of mutations in various genes [4]. The identification of CRC-related genes may help facilitate the early diagnosis, prevention and treatment of the disease [5].

The *TP53* tumor suppressor gene, which is located on chromosome 17p13, is one of the most frequently mutated in human carcinogenesis [6]. The encoded TP53 protein is a key mediator in many cellular processes, including cell cycle arrest, apoptosis, senescence, DNA repair, and changes in metabolism [7]. Consequently, *TP53* mutations may result in a loss of the protein's tumor suppressor function and thus contribute to the development of malignant tumors. The common *TP53* Arg72Pro polymorphism (rs1042522) at codon 72 of exon 4 is the most studied polymorphism in cancer [8]. The guanine to cytosine (G>C) nucleotide exchange associated with this polymorphism leads to a nonsynonymous amino acid change from arginine to proline. The 72Arg variant of TP53 exhibits enhanced ability to localize to the mitochondria and induce apoptosis, whereas the 72Pro variant more efficiently induces cell cycle arrest [9].

Several studies have been conducted to investigate the association between the *TP53* Arg72Pro polymorphism and CRC. However, the results are inconsistent and conflicting. The present meta-analysis was performed to provide a more precise estimation of this association.

## RESULTS

### Study characteristics

**Figure 1:**
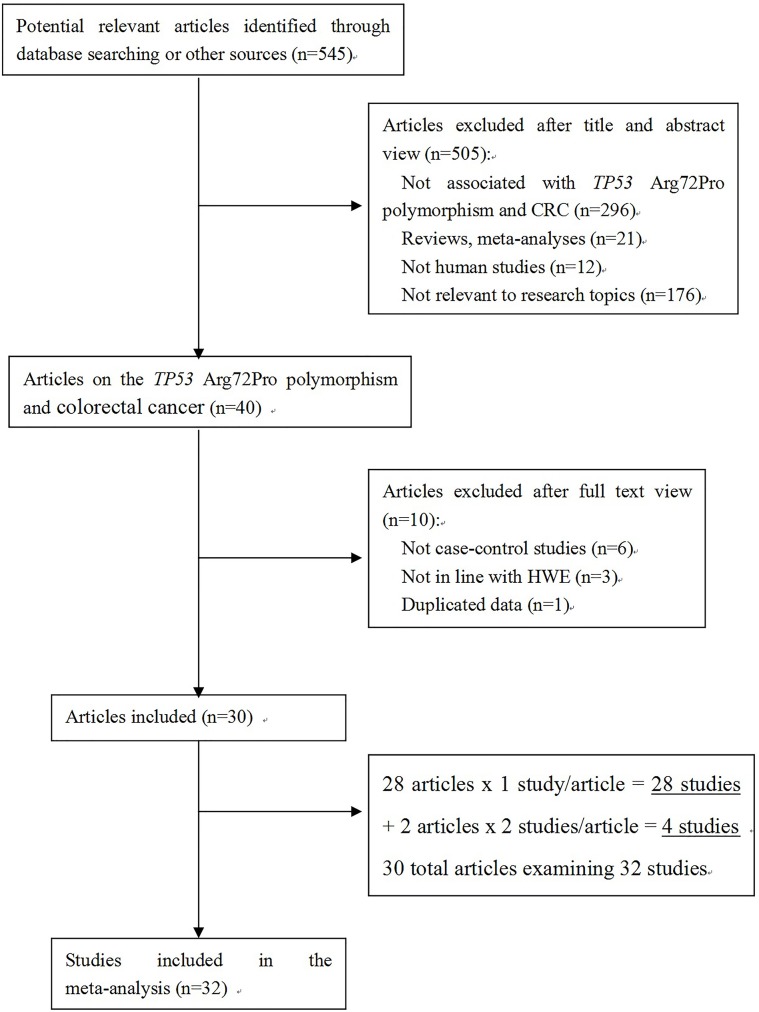
Flow chart of study selection process

Our search strategy yielded a total of 545 records, which were screened to identify original research articles pertaining to TP53 and CRC. The literature search and detailed selection procedures are summarized in Figure 1. After the primary screening, the full text of 40 articles was retrieved for further assessment [10–49]. Ten of those articles were then excluded from further analysis: 6 were not case-control studies [40–45], 1 was based on duplicate data from another eligible study [46], and 3 reported a genotype distribution among the controls that was not in Hardy-Weinberg equilibrium (HWE) [47–49]. Two of the articles reported 2 studies each [19, 24]. Thus, 1 study in each of 28 articles and 2 studies in each of 2 articles, adds up to a total of 32 studies in 30 articles [10–39]. In these 32 studies that conformed to our inclusion criteria, there were 8586 CRC cases and 10275 controls. Fourteen studies involved Asian participants, 12 involved Caucasians, and 6 involved mixed populations. The population characteristics of the included studies are shown in Table 1.

**Table 1:** Characteristics of the individual studies included in the meta-analysis

First author	Year	Country	Ethnicity	Source of control	Type of CRC	Cases			Controls			HWE	Methods
						GG	GC	CC	GG	GC	CC		
Olschwang^10^	1991	France	Caucasian	Population-based	Sporadic	32	34	5	49	52	14	0.97	PCR-RFLP
Kawajiri^11^	1993	Japan	Asian	Population-based	Sporadic	36	32	16	144	165	38	0.36	Allele specific PCR
Murata^12^	1996	Japan	Asian	Hospital-based	Sporadic	46	55	14	53	76	23	0.62	Allele specific PCR
Wang^13^	1999	China	Asian	Hospital-based	Sporadic	18	33	10	43	70	27	0.86	PCR-RFLP
Sayhan^14^	2001	Turkey	Mix	Population-based	Sporadic	26	30	11	21	43	12	0.20	PCR-RFLP
Hamajima^15^	2002	Japan	Asian	Hospital-based	Sporadic	58	72	17	91	107	43	0.24	Allele specific PCR
Gemignani^16^	2004	Spain	Caucasian	Hospital-based	Sporadic	201	133	18	202	95	19	0.09	Allele specific PCR
Schneider^17^	2004	Germany	Caucasian	Population-based	Sporadic	26	26	5	38	41	6	0.25	PCR-SSCP
Krüger^18^	2005	Germany	Caucasian	Population-based	Hereditary	180	95	18	150	78	17	0.13	PCR-RFLP
Sotamaa^19^	2005	Finland	Caucasian	Population-based	Hereditary, Sporadic	231	129	19	172	125	26	0.62	PCR-SSCP
		USA	Mix	Population-based	Hereditary	21	7	2	64	41	13	0.11	PCR-SSCP
Koushik^20^	2006	USA	Mix	Population-based	Sporadic	228	186	28	498	351	55	0.51	Allele specific PCR
Lima^21^	2006	Brazil	Mix	Hospital-based	Sporadic	56	38	6	58	36	6	0.90	Allele specific PCR
Pérez^22^	2006	Argentina	Mix	Population-based	Sporadic	31	20	2	44	53	12	0.50	Allele specific PCR
Perfumo^23^	2006	Italy	Caucasian	Hospital-based	Sporadic	28	30	2	90	49	7	0.92	PCR-RFLP
Talseth^24^	2006	Australia	Caucasian	Population-based	Hereditary	39	19	3	10	11	0	0.10	Sequencing
		Poland	Caucasian	Population-based	Hereditary	33	19	4	45	28	5	0.82	Sequencing
Tan^25^	2007	Germany	Caucasian	Population-based	Sporadic	312	131	24	343	193	27	0.98	Allele specific PCR
Zhu^26^	2007	China	Asian	Population-based	Sporadic	83	117	85	244	321	105	0.97	PCR-RFLP
Grünhage^27^	2008	Germany	Caucasian	Hospital-based	Hereditary, Sporadic	105	72	14	123	78	19	0.20	PCR-RFLP
Csejtei^28^	2008	Hungary	Caucasian	Population-based	Sporadic	66	32	4	62	29	6	0.31	Allele specific PCR
Cao^29^	2009	Korean	Asian	Population-based	Sporadic	54	67	35	114	140	39	0.70	PCR-RFLP
Polakova^30^	2009	Germany	Caucasian	Hospital-based	Sporadic	327	225	60	326	237	49	0.52	PCR-RFLP
Mojtahedi^31^	2010	Iran	Asian	Population-based	Sporadic	46	63	23	58	77	28	0.78	Allele specific PCR
Aizat^32^	2011	Malaysia	Asian	Hospital-based	Sporadic	70	88	44	75	101	25	0.31	PCR-RFLP
Dastjerdi^33^	2011	Iran	Asian	Population-based	Sporadic	97	101	52	76	113	61	0.14	PCR-RFLP
Engin^34^	2011	Turkey	Mix	Hospital-based	Sporadic	50	41	5	52	42	14	0.24	PCR-RFLP
Joshi^35^	2011	Japan	Asian	Population-based	Sporadic	239	342	104	310	361	107	0.90	PCR-RFLP
Song^36^	2011	Korea	Asian	Population-based	Sporadic	740	844	244	734	776	190	0.48	TaqMan
Zhang^37^	2012	China	Asian	Hospital-based	Sporadic	147	199	98	196	271	102	0.62	MALDI-TOF
Oh^38^	2014	Korea	Asian	Hospital-based	Sporadic	222	247	76	145	218	65	0.25	PCR-RFLP
Singamsetty^39^	2014	India	Asian	Population-based	Sporadic	16	48	39	37	45	25	0.13	Sequencing

*HWE*, Hardy-Weinberg equilibrium; *PCR*, polymerase chain reaction; *RFLP*, restriction fragment length polymorphism; *MALDI-TOF*, Matrix-assisted laser desorption/ionization time-of-flight.

### Meta-analysis results

**Figure 2:**
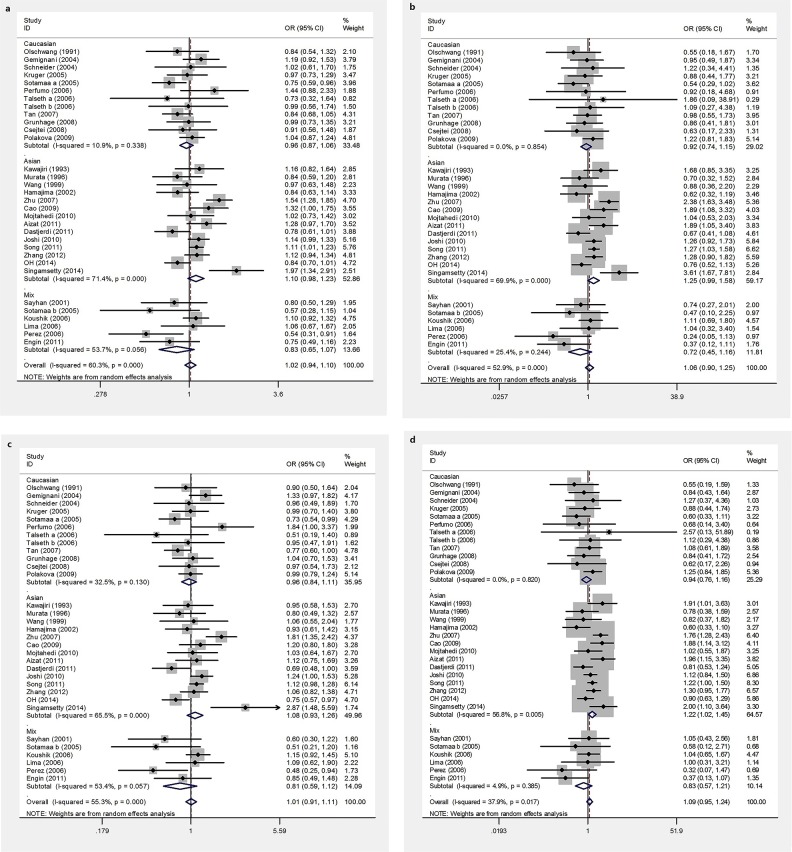
Forest plots of TP53 Arg72Pro polymorphism and CRC risk **a.** allele model, **b.** homozygous model, **c.** dominant models, **d.** recessive models.

We assessed the association between the *TP53* Arg72Pro polymorphism and CRC susceptibility by calculating an odds ratio (OR) and its 95% confidence interval (CI) under the following four genetic models: the allele model (C vs. G), the homozygote model (CC vs. GG), the dominant model (CC+GC vs. GG), and the recessive model (CC vs. GC+GG). A summary of our meta-analysis of the association between the *TP53* Arg72Pro polymorphism and CRC is shown in Table 2. Overall, we observed no significant associations in any of the genetic models (C vs. G: OR =1.02, 95%CI 0.94-1.10; CC vs. GG: OR=1.06, 95%CI 0.90-1.25; CC+GC vs. GG: OR=1.01, 95%CI 0.91-1.11; CC vs. GC+GG: OR=1.09, 95%CI 0.95-1.24) (Figure 2). Further subgroup analyses were conducted to assess the effects of potential confounding factors. There was no evidence for an association between *TP53* Arg72Pro polymorphism and CRC risk in subgroup analyses based on the source of the controls or the type of CRC (Table 2). However, when stratified based on tumor location, we found that the CC genotype increased the risk of rectal cancer (CC vs. GC+GG: OR=1.34, 95%CI 1.12-1.62), but did not alter the risk of colon cancer (CC vs. GC+GG: OR=1.14, 95%CI 0.94-1.39). When the data for rectal cancer were stratified based on ethnicity, no significant associations were observed between *TP53* Arg72Pro polymorphism and CRC risk. Similarly, no associations were found for colon cancer (Table 2). Nonetheless, after stratification based on ethnicity, a significant risk was observed among subjects in Asian populations who carried the CC genotype (CC vs. GC+GG: OR=1.22, 95%CI 1.02-1.45), whereas no risk was observed in Caucasian and mixed populations (CC vs. GC+GG: OR=0.94, 95%CI 0.76-1.16 and OR=0.82, 95%CI 0.5-1.16, respectively). Subgroup analyses based on ethnicity revealed no significant association between *TP53* Arg72Pro polymorphism and CRC risk in Caucasian and Mixed populations.

**Table 2:** Meta-analysis of the association between ***TP53*** Arg72Pro polymorphism and colorectal cancer risk

*Subgroup*	*NO.*	*C vs. G*			*CC vs. GG*			*CC+GC vs. GG*			*CC vs. GC+GG*		
		OR(95%CI)	*P_h_*	*P_OR_*	OR (95%CI)	*P_h_*	*P_OR_*	OR(95%CI)	*P_h_*	*P_OR_*	OR(95%CI)	*P_h_*	*P_OR_*
Overall	32	1.02 (0.94-1.10)	0.000	0.678^*^	1.06 (0.90-1.25)	0.000	0.489^*^	1.01 (0.91-1.11)	0.000	0.912^*^	1.09 (0.95-1.24)	0.017	0.223^*^
Ethnicity													
Caucasian	12	0.96 (0.88-1.05)	0.338	0.359	0.92 (0.74-1.15)	0.854	0.472	0.96 (0.86-1.06)	0.130	0.399	0.94 (0.76-1.16)	0.820	0.555
Asian	14	1.10 (0.98-1.23)	0.000	0.102^*^	1.25 (0.99-1.58)	0.000	0.060^*^	1.08 (0.93-1.26)	0.000	0.300^*^	**1.22 (1.02-1.45)**	0.005^*^	0.026^*^
Mixed	6	0.94 (0.82-1.09)	0.056	0.416	0.79 (0.55-1.12)	0.244	0.181	0.96 (0.81-1.15)	0.057	0.663	0.82 (0.58-1.16)	0.385	0.261
Source of controls													
Population-based	20	1.01 (0.90-1.14)	0.000	0.825^*^	1.12 (0.89-1.41)	0.000	0.319^*^	0.99 (0.85-1.14)	0.000	0.843^*^	1.15 (0.97-1.36)	0.046	0.102^*^
Hospital-based	12	1.01 (0.94-1.09)	0.155	0.744	1.00 (0.85-1.19)	0.165	0.974	1.04 (0.98-1.10)	0.251	0.900	1.04 (0.89-1.21)	0.093	0.636
Tumor location													
Colon cancer	8	1.12 (0.96-1.32)	0.020	0.159^*^	1.23 (0.88-1.73)	0.041	0.228^*^	1.21 (0.94-1.56)	0.005	0.145^*^	1.14 (0.94-1.39)	0.421	0.185
(Caucasian)	2	1.09 (0.90-1.32)	0.456	0.365	1.19 (0.77-1.84)	0.160	0.432	1.11 (0.87-1.41)	0.942	0.410	1.14 (0.75-1.73)	0.104	0.551
(Asian)	5	1.16 (0.86-1.55)	0.003	0.332^*^	1.35 (0.79-2.30)	0.015	0.275^*^	1.29 (0.78-2.13)	0.001	0.319^*^	1.18 (0.93-1.50)	0.414	0.174
(Mixed)	1	1.15 (0.93-1.41)	–	0.192	1.07 (0.61-1.88)	–	0.808	1.25 (0.97-1.62)	–	0.091	0.96 (0.56-1.66)	–	0.888
Rectum cancer	8	1.13 (0.92-1.38)	0.001	0.257^*^	1.36 (0.93-1.99)	0.010	0.108^*^	1.07 (0.83-1.36)	0.018	0.615^*^	**1.34 (1.12-1.62)**	**0.125**	**0.002**
(Caucasian)	2	0.90 (0.72-1.13)	0.549	0.359	1.00 (0.61-1.65)	0.581	0.998	0.82 (0.62-1.09)	0.549	0.161	1.11 (0.68-1.81)	0.687	0.671
(Asian)	5	1.24 (0.93-1.67)	0.001	0.142^*^	1.53 (0.88-2.66)	0.002	0.128^*^	1.24 (0.85-1.79)	0.010	0.264^*^	1.41 (0.97-2.05)	0.034	0.071^*^
(Mixed)	1	1.09 (0.78-1.53)	–	0.626	1.42 (0.64-3.15)	–	0.387	1.03 (0.68-1.58)	–	0.877	1.44 (0.66-3.11)	–	0.360
Type of CRC													
Sporadic	28	1.03 (0.95-1.12)	0.000	0.459^*^	1.09 (0.92-1.29)	0.000	0.323^*^	1.02 (0.92-1.14)	0.000	0.695^*^	1.11 (0.97-1.27)	0.018	0.122^*^
(Caucasian)	9	0.97 (0.88-1.07)	0.069	0.594	0.97 (0.76-1.24)	0.904	0.803	0.96 (0.85-1.09)	0.077	0.540	0.99 (0.78-1.25)	0.838	0.928
(Asian)	14	1.10 (0.98-1.23)	0.000	0.102^*^	1.25 (0.99-1.58)	0.000	0.060^*^	1.08 (0.93-1.26)	0.000	0.300^*^	**1.22 (1.02-1.45)**	0.005^*^	0.026^*^
(Mixed)	5	0.97 (0.84-1.11)	0.299	0.072	0.81 (0.56-1.17)	0.186	0.262	0.99 (0.83-1.19)	0.075	0.923	0.84 (0.59-1.19)	0.287	0.328
Hereditary	6	0.86 (0.73-1.01)	0.422	0.072	0.69 (0.45-1.04)	0.374	0.078	0.87 (0.71-1.06)	0.465	0.158	0.71 (0.47-1.07)	0.417	0.106
(Caucasian)	5	0.88 (0.75-1.05)	0.474	0.148	0.71 (0.46-1.10)	0.284	0.124	0.89 (0.73-1.10)	0.551	0.290	0.73 (0.48-1.11)	0.300	0.141
(Mixed)	1	0.57 (0.28-1.16)	–	0.118	0.47 (0.10-2.25)	–	0.344	0.51 (0.22-1.20)	–	0.123	0.58 (0.12-2.71)	–	0.486
Genotype methods													
PCR-RFLP	14	1.04 (0.92-1.18)	0.000	0.519^*^	1.07 (0.81-1.40)	0.000	0.634^*^	1.04 (0.88-1.23)	0.001	0.628^*^	1.10 (0.89-1.36)	0.011	0.381^*^
Allele specific PCR	10	0.98 (0.89-1.07)	0.153	0.636	0.93 (0.74-1.16)	0.453	0.518	0.98 (0.88-1.11)	0.119	0.791	0.94 (0.76-1.169)	0.348	0.543
PCR-SSCP	3	0.77 (0.62-0.95)	0.383	0.013	0.61 (0.36-1.03)	0.510	0.065	0.74 (0.57-0.95)	0.514	0.020	0.68 (0.41-1.14)	0.559	0.142
Sequencing	3	1.20 (0.65-2.22)	0.031	0.554^*^	2.66 (1.39-5.08)	0.328	0.003	1.18 (0.44-3.12)	0.009	0.745^*^	1.85 (1.08-3.16)	0.731	0.025

*OR* odds ratio; *95%CI* 95% confidence interval; *P_OR_*, pool *P* value; *P_h_*, *P* value of heterogeneity test;

* Estimates for random-effects model; otherwise, fixed-effects model was used.

### Publication bias and sensitivity analysis

**Figure 3:**
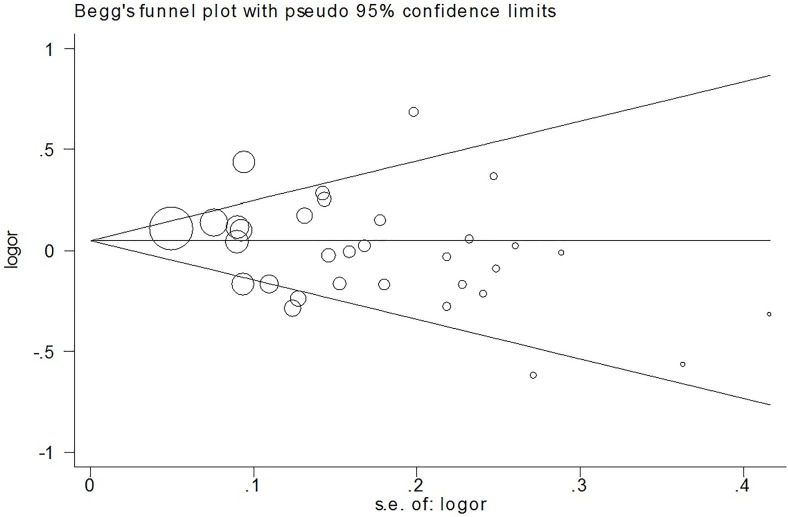
Beggar's funnel plot of TP53 Arg72Pro polymorphism and CRC risk under the allele model

We used Begg's funnel plot and Egger's test to assess the publication bias of the published articles. The symmetrical funnel plot for the allele model shown in Figure 3 suggests the findings of our meta-analysis were not affected by publication bias. The Egger's test results also did not suggest the existence of publication bias, as indicated by *P* values greater than 0.05 (*P*=0.098 for the allele model). The influence of each individual study on the pooled OR was assessed by performing the analysis while deleting one study at a time. Because the OR was not significantly influenced by omitting any single study (data not shown), we conclude our data are relatively stable and credible.

## DISCUSSION

The mechanisms that underlie the development of CRC are complex, and both environmental and genetic factors play important roles in the occurrence and progression of this disease [50]. TP53 is crucial for proper control of gene transcription, DNA synthesis and repair, cell cycle arrest, senescence and apoptosis. Mutations in *TP53* can disrupt these functions, leading to genetic instability and the progression to cancer.

In this meta-analysis, we found that the *TP53* Arg72Pro polymorphism was not associated with CRC in patients stratified based on type of CRC, genotype method or source of controls. When stratified based on ethnicity, there was a positive association between the *TP53* Arg72Pro polymorphism and CRC risk in Asian populations, but not Caucasian or mixed populations. These differences may reflect differences in genetic background and/or environmental factors. The Arg72 variant of the *TP53* Arg72Pro polymorphism is more efficient with respect to mitochondrial localization than the Pro72 variant and has a stronger capacity to induce apoptosis [51]. Researchers observed that the Arg72 form induced apoptosis with faster kinetics than did the Pro72 variant [52]. The greater apoptotic potential of the Arg72 protein stems from the greater interaction of this protein with MDM2, which facilitates nuclear export [53]. The two polymorphic variants of TP53 are functionally distinct, and these differences may influence cancer risk or treatment. Our result is does not confirm the findings of 2 earlier meta-analyses [54, 55]. These differences may be the result of the rigid inclusion criteria of our study. We excluded two studies with control genotypic distributions that deviated from the HWE [47, 48] and 2 studies with overlapping populations [18, 46]. We also identified 8 studies as eligible [32–39] that were not included in earlier meta-analyses. Thus, our meta-analysis likely provides a more precise estimate of the relationship between the *TP53* Arg72Pro polymorphism and CRC risk.

Several studies have indicated that there are multiple differences in the epidemiological, pathological and molecular features of CRCs [56–58]. Kapiteijn et al. indicated that rectal cancer may involve more nuclear β-catenin in the APC/β-catenin pathway than colon cancer and reported that the p53-pathway also appears to be more important in rectal cancer [57]. In another study, Slattery et al. found that rectal and distal colon tumors are more likely to have a p53 mutation than proximal colon tumors [58]. When we stratified based on tumor location, we observed a significant association between the *TP53* Arg72Pro CC genotype and rectal cancer, but no association was observed between this genotype and colon cancer. One possible explanation for this finding could be that different bacterial flora and a longer transit time in the rectum might change the contact between intestinal cells and potential carcinogens or promoters in the fecal stream, which may lead to more (exogenous) mutations of p53.

Factors known to affect the risk of CRC include gender, age, environmental factors and chronic inflammation. Joshi et al. found that men with the CC genotype and C allele had significantly higher risk for CRC than women with the same genotype [35]. Aizat et al. found that carriers of CC genotype aged 50 years and older were also at significantly greater risk for CRC [32]. However, no significant associations were found between these two confounding factors and CRC susceptibility in other studies [26, 29]. The difference may be explained by differences in the groups studied or populations and/or by differences in environmental exposure and lifestyle factors. Additional studies with a large patient cohort are needed to verify these initial observations.

Our meta-analysis had several limitations. First, we did not calculate an adjusted estimate for the association between the *TP53* Arg72Pro polymorphism and CRC risk because not all studies reported adjusted ORs. Second, because heterogeneity was obvious, even in some sub-analyses, other potential confounding factors appeared to be present in the included studies; we did not take these confounding factors into account. Third, due to an absence of information, we were unable to assess other factors such as gender, age, alcohol consumption and smoking status, which may have modified the association. Finally, potential gene-gene and gene-environment interactions were not analyzed due to a lack of relevant data.

In summary, our updated meta-analysis demonstrated that the *TP53* Arg72Pro polymorphism CC genotype may contribute to an increased risk of CRC, especially for rectal cancer and among Asians. Future well-designed studies with larger samples are needed to confirm our findings.

## MATERIALS AND METHODS

### Identification of eligible studies

Potentially relevant articles published prior to December 2014 were identified in the PubMed, EMBASE, Web of Knowledge, and Chinese National Knowledge Infrastructure databases using the following key words: “TP53 or P53,” “polymorphism or variant,” and “colorectal cancer, colon or CRC.” Additional studies on the topic of interest were identified by hand-searching the reference lists of the retrieved articles. When multiple publications reported on the same or overlapping data, the most recent study with the largest sample size was selected.

### Inclusion and exclusion criteria

The studies included in our meta-analysis were required to meet the following criteria: 1) the study was a case-control or cohort study; 2) the study investigated the association between the *TP53* Arg72Pro polymorphism and CRC risk; 3) the study provided sufficient information to estimate ORs and 95% CIs; and 4) the study had a control genotype distribution in HWE. Studies were excluded for the following reasons: 1) the study was not a case-control study; 2) the publication contained incomplete data; and 3) the study was a duplicate of a previous publication.

### Data extraction

Data were independently extracted by two reviewers (Dai and Sun) using a standardized data extraction form. Disagreements were resolved through discussion. The extracted data included the following items: first author, publication year, country of origin, ethnicity, source of control, sample sizes, genotype distribution in cases and controls, *P*-value for HWE, and genotyping methods.

### Statistical analysis

Pooled ORs with corresponding 95% CIs were used to evaluate the strength of the observed associations. Four genetic contrast models, including allelic contrast (C vs. G), homozygote comparisons (CC vs. GG), dominant models (CC+GC vs. GG), and recessive models (CC vs. GC+GG), were applied. HWE was evaluated in the control group for each study using the χ^2^ test, and the significance level was set at *P*<0.05. Between-study heterogeneity was assessed by calculating the *Q*-statistic and quantified using the *I^2^* value. A fixed effect model that used the Mantel-Haenszel approach was applied to calculate the pooled ORs if the between-study heterogeneity was not significant [59]. A random effect model that used DerSimonian and Laird's method was adopted when the between-study heterogeneity was obvious [60]. When the Q test *P*>0.05 and *I^2^*<50%, the fixed-effects model was used; otherwise, the random-effects model was used. Subgroup analyses were performed based on ethnicity, source of controls, tumor location and genotype method. Sensitivity analysis was performed to determine the influence of single datasets on the combined estimates. Begg's funnel plot and Egger's test were used to assess publication bias [61, 62]. All analyses were performed using Stata software version 12.0 (Stata Corp., College Station, TX), and all *P* values were two-sided.
